# Salinity and temperature increase impact groundwater crustaceans

**DOI:** 10.1038/s41598-020-69050-7

**Published:** 2020-07-23

**Authors:** Andrea Castaño-Sánchez, Grant C. Hose, Ana Sofia P. S. Reboleira

**Affiliations:** 1 Natural History Museum of Denmark, University of Copenhagen, Universitetsparken 15, 2100 Copenhagen, Denmark; 2 Department of Biological Sciences, Macquarie University, Sydney, NSW 2109 Australia

**Keywords:** Ecology, Climate sciences, Environmental sciences, Limnology

## Abstract

Anthropogenic impacts in groundwater ecosystems remain poorly known. Climate change is omnipresent, while groundwater salinization poses serious long-term environmental problems in arid and semi-arid regions, and is exacerbated by global warming. Both are present threats to the conservation of groundwater ecosystems, which harbour highly specialized species, with peculiar traits and limited geographic distributions. We tested the temperature and salinity tolerance of groundwater-adapted invertebrates to understand the effect of global warming and salinization in groundwater ecosystems. We used species representative of groundwater-adapted crustaceans: two copepods (harpacticoid and cyclopoid) and one syncarid, endemic to Australia. Our results show that 50% of the populations died at salt concentrations between 2.84 to 7.35 g NaCl/L after 96 h, and at 6.9 °C above the ambient aquifer temperature for copepods and more than 10 °C for syncarids. Both copepods were more sensitive to temperature and NaCl than the syncarid. We calculated a salinity risk quotient of 9.7 and predicted the risk of loss of 10% of syncarid and 20% of copepod population abundances under a worst-case scenario of global warming predictions for 2070. These results highlight that both salinity and temperature increases pose a risk to the ecological integrity of groundwater ecosystems.

## Introduction

Groundwater comprises 97% of the freshwater global resources available for direct human consumption^1^. It harbours a unique ecosystem, composed of groundwater-adapted species, mostly crustaceans, which provide ecosystem functions linked with nutrient recycling and water purification^2^. The biota, and their ecosystem services, are under increasing threat from anthropogenic activities causing changes to the aquifer environment^3^.

Salinization is a process in which the mobilization and/or fractionation of salts causes an increase salt concentration in water and soils^4^. Human activities can induce salinization of groundwater in multiple ways, such as seawater intrusion into coastal aquifers caused by rising sea levels and excessive groundwater pumping^5,6^, inland salinization caused by rising and lowering the water tables through saline sediment layers^4^ or by direct salt application, e.g., to prevent ice formation on roads^7^. Salinization processes associated with human activities are notably intensified in arid and semi-arid regions which, at the same time, are expected to be very sensitive to climate change effects, particularly, increased temperatures due to global warming^8,9^.

Australia has the world’s highest proportion of salt-affected soils, and with that, the sum of salts in groundwaters reach as high as 19.32 g/L^10,11^ (sea salt concentrations around 30 g/L^10^). Large increases in salinity (e.g., EC ranged from 7,000 to 27,000 µS/cm or 600 to 17,000 µS/cm) have been observed over the last 50 years, especially in shallow aquifers^11^. High salinities in some parts of Australia are due to the geological history of the continent, much of which was subject to periods of marine inundation which have left saline layers in the geological profile^12^, but high salinities may be also due to dryland salinity, a consequence of land clearing, over grazing and irrigation^10,11,13^. While sodium chloride (NaCl) is a common component of natural waters, increasing concentrations of NaCl and other salts in groundwater are a serious long-term environmental problem, that impacts (among others) surface vegetation (including agricultural crops), groundwater-dependent ecosystems and drinking water quality^4^, as well as roads and built infrastructure. All of these impacts may be exacerbated with global warming^9^. The ability of organisms to tolerate salinity (stenohaline vs. euryhaline) is related to how they deal with the osmotic stress^14^, which, for groundwater organisms, remains largely unknown.

As for salinity, little is known of how temperature increases impact upon groundwater species^2,15^. Groundwater organisms live in very stable thermal conditions and because of this, they may be particularly threatened by temperature increases (e.g., climate change) that will likely increase the metabolic rates, food and oxygen demand^16^. It is predicted that by 2070, the annual average temperatures in Australia may increase by 1.0–6.0 °C^17^, which is expected to be reflected in groundwater temperatures that follow the mean annual surface temperatures^18^.

The biota of groundwater ecosystems is unique and different to that found in even connected surface waters. Groundwaters lack photosynthetic organisms, trophic chains are typically short and its fauna (collectively ‘stygofauna’) is dominated by highly endemic crustaceans, which are considered to be particularly vulnerable to anthropogenic impacts^1,19,20^. Therefore, it is critical to understand how groundwater adapted fauna (stygobionts) respond in order to develop realistic assessments of the risks of increasing salinities and temperatures on groundwater ecosystems. The aim of this study is therefore to estimate the effects of salinization and temperature increases on specialized groundwater crustaceans. We estimate experimentally the upper thermal tolerance and the acute lethal salinity concentration for three groundwater-adapted crustaceans, and predict their extinction risk under global warming scenarios, and for measured environmental salinity concentrations.

## Results

### Acute salinity tolerance test

**Figure 1:**
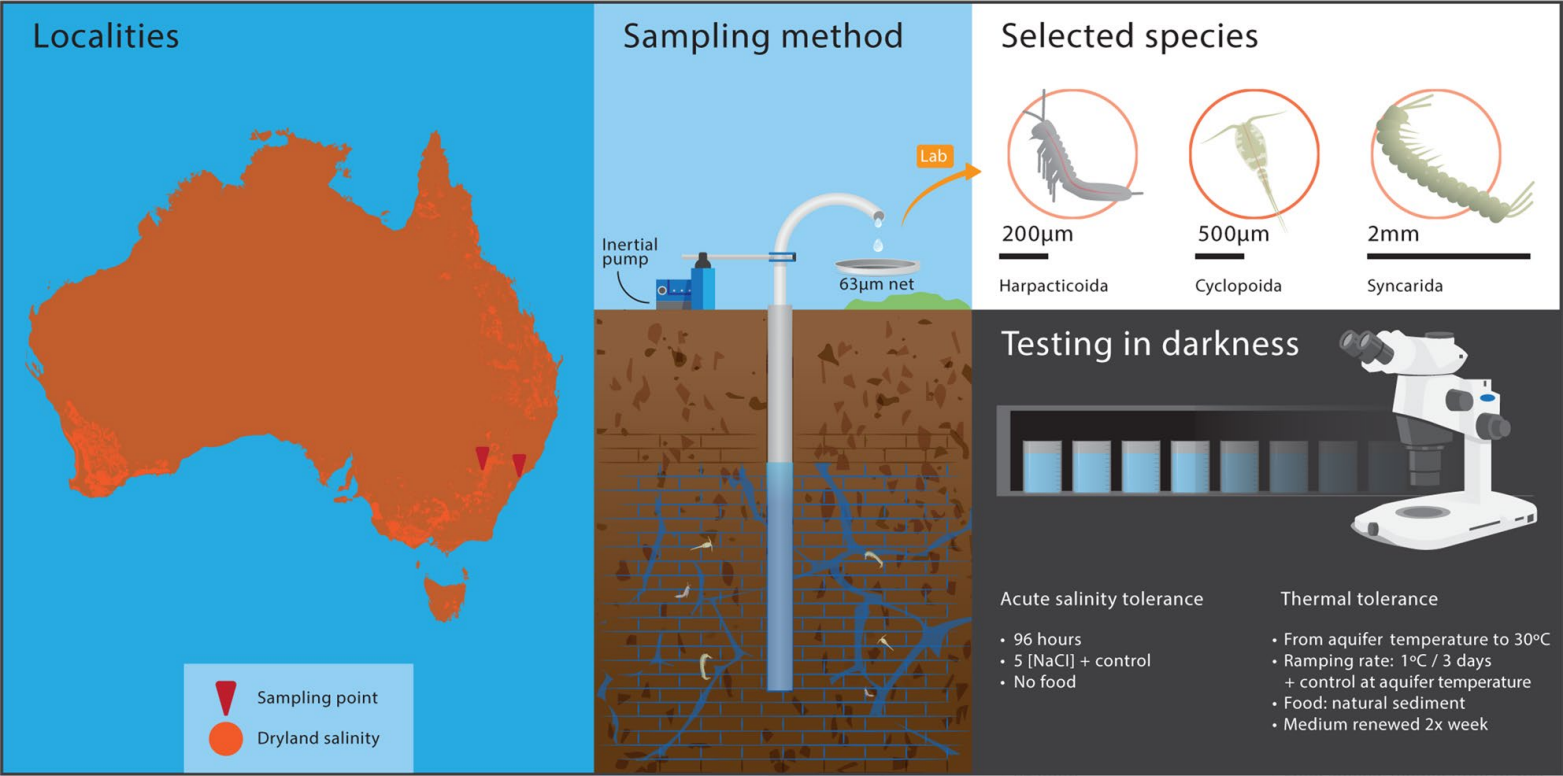
Graphical representation of the experiment design.

**Figure 2:**
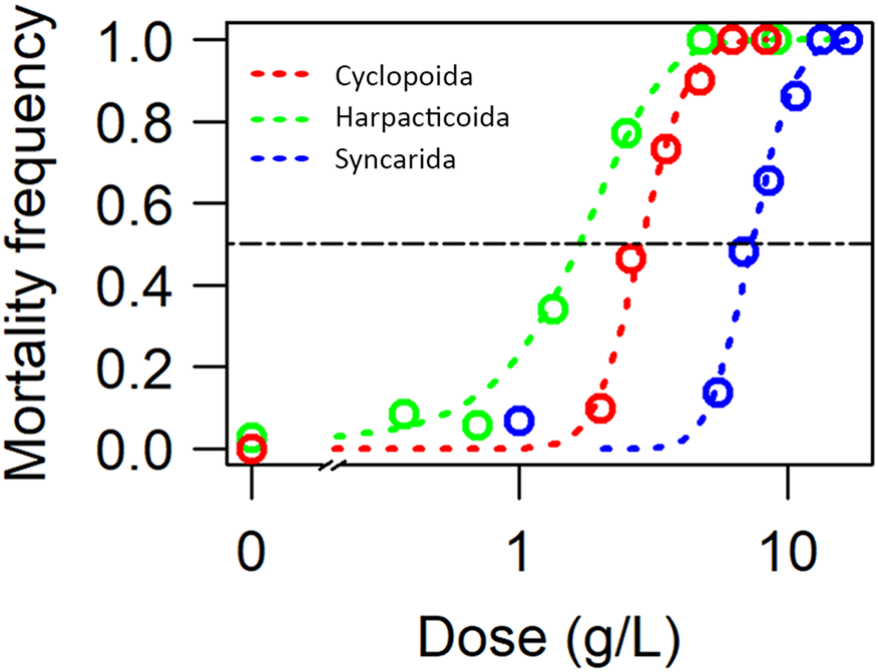
NaCl dose–response curves and median lethal concentrations (LC_50_) at 96 h.

The syncarid was the most tolerant of the taxa tested (96 h LC_50_ = 7.35; LC_10_ = 5.05 g NaCl/L) (Table 1). The cyclopoid copepod (96 h LC_50_ = 2.84; LC_10_ = 1.88 g NaCl/L) (Table 1) was more tolerant than the harpacticoid (LC_50_ = 1.67; LC_10_ = 0.58 g NaCl/L). The cyclopoid copepod and the syncarid had a narrower mortality range than the harpacticoid (Figs. 1 and 2).

**Table 1:** LC_50_ and LC_10_ values of groundwater-adapted crustaceans at 96 h and 95% confidence intervals.

Taxa	LC_50_ (NaCl g/L)	LC_10_ (NaCl g/L)
Harpacticoida	1.67 (0.65–2.68)	0.58 (0.30–0.87)
Cyclopoida	2.84 (1.31–4.37)	1.88 (0.50–3.20)
Syncarida	7.35 (6.80–7.90)	5.05 (4.33–5.77)

### Environmental risk assessment

**Figure 3:**
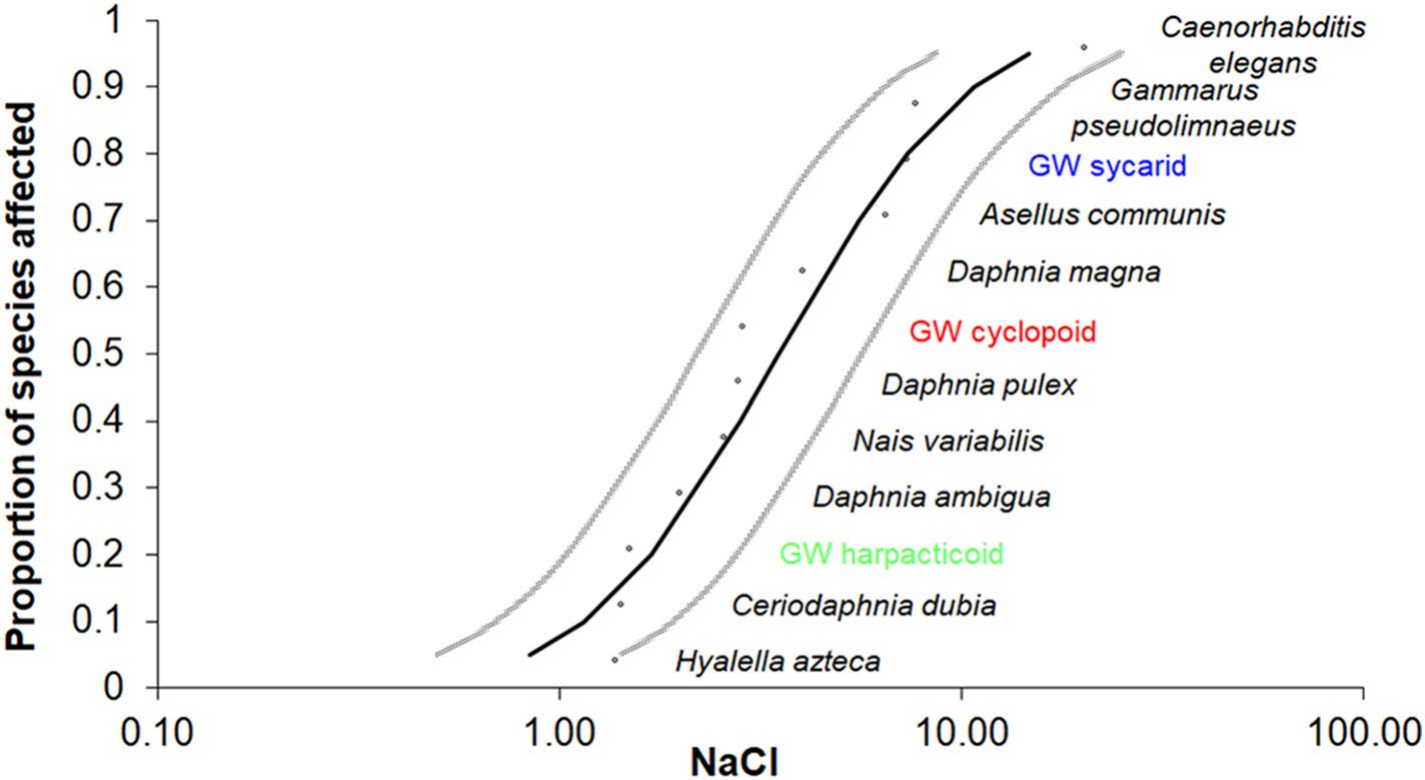
Species sensitivity distribution for salt (NaCl, g/L) based on the LC_50_ of the tested species and freshwater organisms with 95% confidence intervals.

From the species sensitivity distribution (SSD) curve (Fig. 3, Supplementary Table S1) performed with the LC_50_ values obtained in this study for groundwater-adapted crustaceans and the acute data of freshwater crustaceans as surrogate (Supplementary Table S2), we obtained the 5% hazardous concentration (HC_5_) of 0.841 (1.426–0.496) g NaCl/L. This value was used as a predicted no effect concentration (PNEC) to estimate the risk quotient (RQ). We obtained a salinity RQ of 9.70, which implies an environmental risk for the study area.

### Upper-thermal tolerance

**Figure 4:**
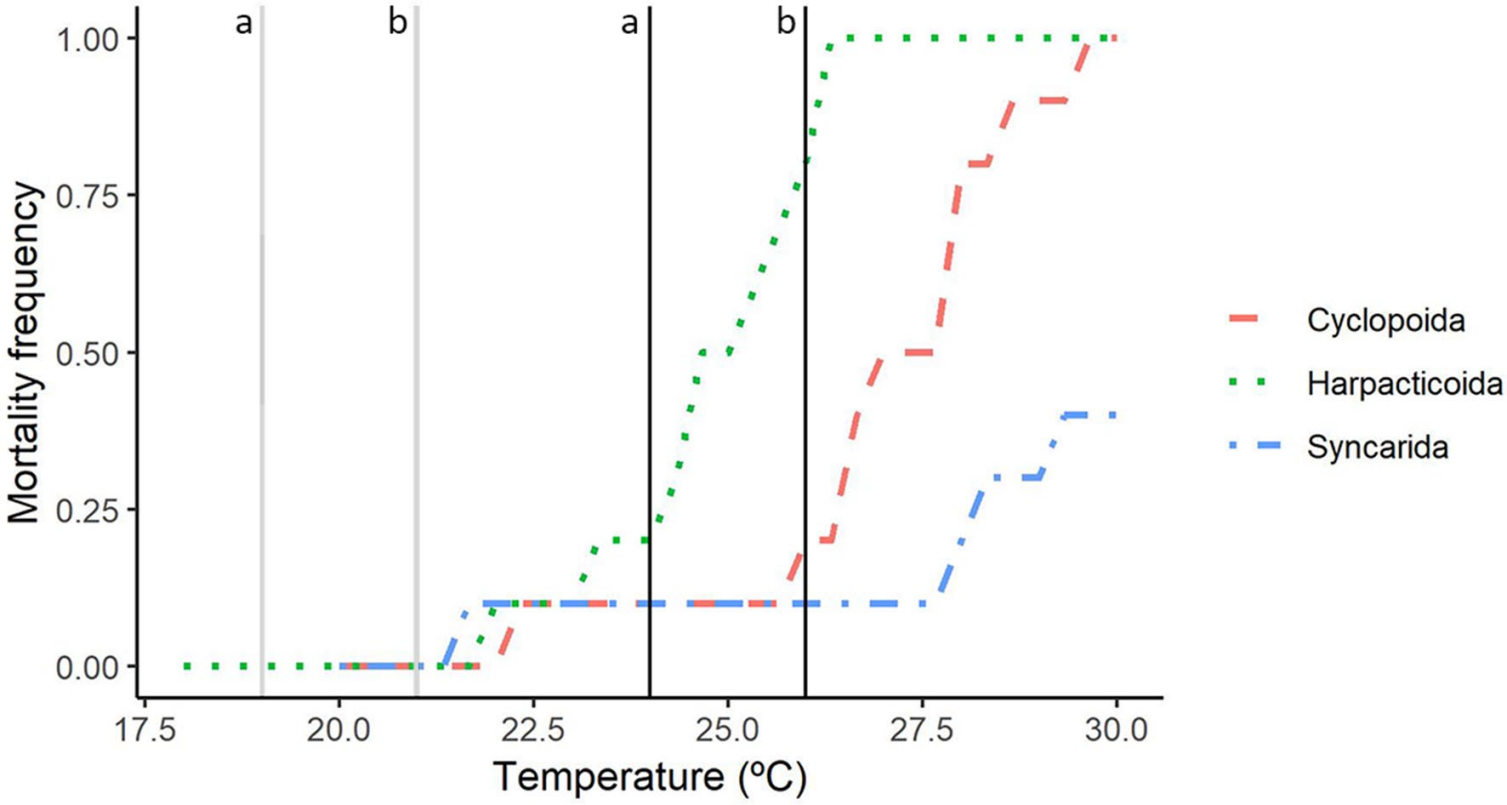
Upper thermal tolerance and predicted temperatures for each aquifer by 2070 (Somerby aquifer correspond to the line “a” and Wellington aquifer to the line “b”) using the best scenarios (grey lines) and the worst scenarios (black lines).

The syncarid was more tolerant to temperature increases than were the copepods. More than 60% of the syncarids were still alive at 30 °C meaning that the upper thermal limit at which 50% of the populations died (UTL_50_) was > 30 °C (Figs. 1 and 4). The cyclopoid copepod (UTL_50_ = 26.9 ± 0.2 °C) was more tolerant to the temperature increases than was the harpacticoid (UTL_50_ = 24.8 °C ± 0.2 °C), however, the UTL_50_ of both copepod species were 6.9 °C above their measured environmental concentrations.

## Discussion

In contrast to fresh surface waters where insects dominate the invertebrate biodiversity, groundwaters are dominated by crustaceans^21^. Little is known of the responses of groundwater-adapted species to climate change and nothing was previously known on their responses to salinity increases^2^.

Sensitivity to increases of NaCl and temperature among the tested species followed the same patterns, with the harpacticoids being the most sensitive to both stressors, followed by cyclopoids, and syncarids. This general pattern matches with the salinity and temperature from which the organisms were collected; the Macquarie River alluvial aquifer (Wellington) from which the syncarids and cyclopoids were collected had higher salinity and temperature than the Somersby fractured rock aquifer from which the harpacticoids were collected. This difference in tolerance based on collection location suggests either pre-adaption to local conditions or a genuine broader thermal and salinity tolerance in the taxa from Wellington. However, both copepods species had UTL_50_ values that were 6.9 °C above their aquifer ambient temperature suggesting that thermal tolerance is independent of, and thus not a pre-adaptation to, warmer thermal regimes.

The relatively lower sensitivity of syncarids to temperature and salinity may be linked to their larger body size compared to the copepods. Body size is related to the organisms’ energetic demand, which is also associated to the metabolic rate and feeding activity^22^. However, there was no size-dependent sensitivity trend when we analyzed responses of freshwater crustaceans to salinity. The most sensitive species in the SSD curve (Fig. 3) was the widely distributed amphipod *Hyalella azteca,* which is considerably larger (3–8 mm length) than the groundwater copepods studied here and the freshwater cladocerans also used in the SSD^23^. The sensitivity of freshwater Cladocera to a range of stressors is negatively related to body size, independent of their geographical distributions^24,25^. However, there was no significant relationship between body size and sensitivity to organic pollutants in freshwater and marine Branchiopoda, Copepoda and Ostracoda^26^. Thus, within the non-cladoceran aquatic crustaceans, physiology, metabolism and respiratory activity (tissue oxygen supply and aerobic scope) seem to play a greater role in determining species tolerance to stressors than body size^26,27^.

Despite the considerable knowledge of the hydrology and geochemistry of areas affected by inland salinization in southeastern Australia^11^, biological studies are scarce and the ecotoxicological effects of salinization to groundwater biota has until now been neglected. Studies using freshwater organisms highlighted a lower abundance and richness of microinvertebrates in the areas with the highest salinity ^28,29^, while in groundwater, biomonitoring studies in aquifers along a salt-gradient, have shown relatively low abundance of groundwater-adapted species (expressed as the ratio of stygoxene/stygobiont spp) in boreholes with higher salinity^29,30^.

The MEC value (8.259 NaCl g/L) used for our risk assessment was based on available salinization data for southeastern Australia^10^. Our HC_5_ values were calculated using a probabilistic approach without the application of an assessment factor (AF) which are often applied to such values to account for the use of acute laboratory data. In this case, the use of an AF reduced the HC_5_ value below the background concentration of NaCl in groundwaters, and was thus overly conservative. Nevertheless, the resulting risk quotient was greater than 1, even without the application of an AF, which is a particular cause for concern and suggests that current salinity levels in that region pose a potential risk to groundwater invertebrates. Interestingly, in other regions of Australia (e.g., in the border rivers catchment of the Murray-Darling Basin), unidentified species of groundwater-adapted harpacticoids, cyclopoids and syncarids have been reported living at salinities much higher than our MEC value (22.5–32.5 g/L of dissolved solids)^31^. Since stygobitic crustaceans are present in groundwaters with a broad range of salinities, region-based risk assessments are clearly needed, following approaches similar to those proposed for metal contamination^32^.

In this study, the syncarid had a wider tolerance to heat (< 40% mortality at more than 10 °C above the measured environmental temperature) than the two copepods species (50% mortality at 6.9 °C above the measured environmental temperature). Data on the upper thermal tolerance of groundwater crustaceans is crucial to predict their survival under climatic changes, where an increase in temperatures is expected for the usually thermally stable groundwater environments^15^. This is particularly important given that marine benthic invertebrates from thermally stable Antarctic and tropical environments were less tolerant to temperature increase (under long warming rate experiments) than temperate species, which are usually subjected wide ranges of temperature^33^. The temperature tolerance range of groundwater crustaceans is poorly known. The limited available data shows that the amphipod *Niphargus rhenorhodanensis* survived at temperatures between − 2 and 28 °C, that the isopod *Proasellus valdensis* had 90% survival between 2 and 16 °C, and mortality of the isopod *P. cavaticus* increased rapidly when temperatures varied from 10 °C^34,35^. Only the stygobiont *P. cavaticus* had a very narrow thermal tolerance (stenothermic)^35^. The scarcity of thermal tolerance data for groundwater animals, paired with the variability of methodologies used (i.e., acclimation, rate of increase, experimental conditions, and endpoints measured) preclude direct comparison of results^2,36,37^.

Climate models predict a temperature increase of 1 °C by 2070 in Australia as the best-case scenario^17^. Our results suggest that such a change will not pose a risk the tested organisms since no mortality was observed following this increase. However, the increase of 6 °C predicted under the worst scenario^17^ will pose a major risk to all three species tested, where 20% of the copepods populations and 10% of the syncarid population are likely to die. Prior to this study, only a single study was conducted to evaluate the effect of climate change on groundwater organisms in which it was observed that the expected increase in the annual mean temperature of 3 °C in the Mediterranean region by 2050 should not cause any change in the metabolic activity of the copepod *Diacyclops belgicus*^15^*.* Although, slow temperature ramping had been used to test the thermal tolerance of groundwater organisms (e.g., 1 °C every four days^35^), the use of more realistic and ecologically relevant warming rates was recommended^27^, because the average warming rate expected for the predicted worst case scenario in the study area corresponds to a very low rate of 0.01 °C/month. Therefore, the acclimation period expected in the aquifer is around 1,000 times slower than the warming rate used in laboratory studies. However, this more gradual rate of increase may lead to greater mortality, at least in the short term. This is supported by the oxygen and capacity limitation of the thermal tolerance hypothesis^38^, which proposes that under short-term exposures with high ramping rates, death may be delayed by the use of compensatory anaerobic metabolism, which enables organisms to tolerate temperatures beyond that which aerobic metabolism would allow^27^. For example, a considerable decrease in survival was observed when the acclimation period was increased from 1 °C day to 1 °C month in Artic marine ectotherms, where at the slowest ramping rates, species died 2 °C or 3 °C above current summer maximum temperatures^27^.

While global warming is indisputably a serious problem, there are also other anthropogenic causes for temperature fluctuations in groundwater, which can, on a local scale, be much more pronounced (with faster warming rates). For example, the groundwater temperatures underneath large cities can be up to 5 °C higher than the temperature of the surrounding aquifer^39^, due to the continuous heat discharge from district heating and public sewer networks. In addition, when heat is being actively stored in aquifers, e.g., for cooling purposes, temperatures ≥ 30 °C are often observed^40^.

As long foreseen in surface environments^41^, global warming and salinization also present a concerning picture for groundwater ecosystems. Due to their relic condition, many groundwater species (e.g., the tested Bathynellacea syncarid) lack closely related surface-dwelling species, because ancestors became extinct due to climatic or catastrophic events^42^. The unique traits of groundwater species (long life cycles, reduced fertility, small populations size, and short distributions) make the recovery and recolonization impossible or extremely reduced and increases the extinction risk of groundwater species in case of disturbance^3^. The current knowledge of thermal and chemical stressor impacts on groundwater fauna are currently inadequate for establishing robust thresholds for environmental protection and supporting policy that will ensure the preservation of groundwater ecosystems. Further research should target the interactive effects of temperature and salinity, and of other pollutants, because it is known that salinization enhances the toxicity of organic pollutants in freshwater organisms^43,44^, and the higher temperatures increases the rate of pollutants uptake due to the increase of physiological activity and decrease in oxygen solubility^45,46^.

## Material and methods

### Sampling, acclimation and taxa selection

Specimens were collected from groundwater via boreholes using a motorised inertial pump (Waterra, ON, Canada), placed in a sealable 1-L plastic container and filled with local groundwater for transportation to the laboratory in a portable cooler (Fig. 1). Dryland salinity map was produced in software QGIS version 3.10.2-A Coruña, using the shapefile Australian Dryland Salinity Assessment Spatial Data (1:2,500,000)—NLWRA 2001 (https://data.gov.au). Figure 1 was drawn in Adobe Illustrator CS6 (Adobe Systems). Temperature, electrical conductivity (EC), pH and dissolved oxygen (DO) concentration in the groundwater at the time of collection were measured using a YSI Plus multimeter (YSI, Ohio USA) (Table 2).

**Table 2:** Sampling localities and physicochemical parameters of groundwater.

Borehole locality	Latitude	Longitude	Depth (m)	Altitude (m)	T (°C)	pH	EC (µS/cm)	DO (mg/L)
Wellington, NSW	32°34′26.78″S	148°59′23.7″E	23	285	19.7	7	1,079	2.9
Somersby, NSW	33°22′15.42″S	151°18′09.0″E	22	248	18.07	4.95	162	5.2

Depth, of the drilled borehole; *T* temperature, *EC* electrical conductivity, and *DO* dissolved oxygen.

In the laboratory, specimens were sorted under a stereomicroscope (Olympus Z16), and acclimated for at least 48 h prior to testing inside plastic containers with groundwater and sediments from the collection site, in a dark environmental cabinet (Labec Pty Ltd, Sydney) at aquifer temperature. The age and gender of the test organisms was unknown at the time of testing – the detailed examination of test organisms prior to testing was avoided to minimise stress on the animals. The random allocation of test animals to treatments avoided specific bias, and all animals used were of a similar size to minimise the likelihood of age differences, and obviously gravid female (i.e., those copepods bearing easily discernible egg sacs) were avoided. All specimens tested are deposited in the Natural History Museum of Denmark. Three species were tested separately. The first was a harpacticoid copepod (Copepoda: Harpacticoida; Ameiridae n. sp.; GenBank accession numbers KF361325, KF361326 and KF361332) that was collected from a fractured sandstone aquifer in Somersby, New South Wales, Australia (Table 2). This species has been used previously in toxicity tests^47^. The second species was a cyclopoid copepod (Copepoda: Cyclopoida: Cyclopidae: *Diacyclops* n. sp.) from the Macquarie River alluvial aquifer at Wellington, NSW, Australia. The third was a parabathynellid syncarid (Syncarida, Bathynellacea, Parabathynellidae n. sp., GenBank accession numbers KF361321 and KF361324) also from the Wellington aquifer^48^. This syncarid has also been used in previous ecotoxicity studies^47^. The three test species belong to two different trophic levels: copepods are primary consumers (feeding on particulate organic matter and bacteria biofilms), while syncarids have an omnivorous diet including predation and cannibalistic activities^49,50^.

### Acute salinity tolerance test

Acute toxicity tests were carried out over 96 h using sodium chloride [CAS]: 7647-14-5 (Sigma-Aldrich, Australia, 99% purity) as the stressor. Acute exposure for the different species followed the standard protocols for surface freshwater species^51–53^ and following the recommended modifications for stygobiotic crustaceans^54^. A geometric range of five treatment concentrations was selected based on preliminary testing (Supplementary Table S3). Tests were conducted using filtered groundwater (0.2 µm pore size sterilized filter, to ensure starvation conditions) from each collection site as a control medium and diluent water. Salinity in the controls correspond to a natural salt concentration of 101.91 mg/L in the borehole from Wellington and while the Somersby aquifer corresponds to freshwater EC values, i.e. not affected by salinity. Treatment concentrations were validated based on electrical conductivity measurements.

A total of 30 specimens (collected in two sampling occasions during a month) per treatment concentration were tested. Live and actively moving individuals were randomly selected from the acclimated population and tested in a final volume of 10 mL. The two species of copepods were tested using five specimens per vial (6 replicates per treatment concentration), following recommendations^54^, while the syncarids were tested individually to avoid cannibalism. Tests were considered valid if control mortality was below 20%^54^.

The tests with harpacticoids were conducted at 18 ± 1 °C and tests for cyclopoids and syncarids were conducted at 20 ± 1 °C, reflecting the conditions at the time and site of collection (Table 2). Tests were conducted in the dark and test animals were not fed during the tests. Each test vial was observed every 24 h for mortality, which was defined as lack of movement or swimming after gentle stimulation by a sorting needle. EC, DO and pH were measured at the beginning and end of the experiment using hand-held meters (Hanna Inc, USA)^54^. Mortality responses at 96 h were used to determine lethal concentration which affect 50% and 10% of the population (LC_50_ and LC_10,_ respectively)_._

### Upper-thermal tolerance test

The test started at the temperature of groundwater at the time and site of collection of each taxon. Temperature was increased by 1 °C every 3 days up to maximum temperature of 30 °C, and the tests were conducted in the dark following methods previously used with subterranean species^34,35,55^. Control organisms were maintained in a controlled temperature room at the starting temperatures (Sherer-Gillete Co., Marshall, Mich, USA). Temperature increases were done using an environmental cabinet (Labec Pty Ltd, Sydney) with the temperature accuracy of ± 0.1 °C.

Experiments were conducted with 10 specimens (collected in a single sampling period) in the temperature change treatment and the control. Test organisms were kept individually in groundwater from the collection locality and a small amount of fine sediment (collected from the aquifer during pumping) to provide a substrate with available food and preserve native physico-chemical conditions. Harpacticoid and cyclopoid specimens were accommodated in 2 mL (+ 15 to 20 mg of sediment), and the larger syncarids in 8 mL (+ 35 mg of sediment). Twice a week, 75% of the groundwater volume was renewed using a sterile pipette, and physico-chemical parameters (temperature, pH and DO concentrations) were measured to validate the test following the acute salinity test procedure. Mortality was recorded every 24 h, using the same criteria as for the salinity test. Mortality responses over time were used to determine upper lethal temperature affecting 50% of the population (UTL_50_)_._

### Statistical analyses

Acute concentration response curves were estimated by fitting a two parameter, nonlinear-regression function using the DRC package version 3.0-1^56^. Weibull, Gompertz, log-logistic and log normal model were tested where the best fitting model for each test was chosen by comparison of Akaikes information criterion^47^. Lethal concentrations (LC_50/10_) were extrapolated from the fitted curve. Data for the UTL was fitted to a generalized linear model (glm) assuming a binomial distribution to fit a curve to the log-transformed data. The upper lethal limit (UTL_50_) was extrapolated from the fitted curve using the MASS package version 7.3–49^57^ applying the *dose.p* function. All analyses were performed in R version 3.5.0^58^.

### Environmental risk assessment

Direct estimation of NaCl effects in southeastern Australia aquifers was based on ecotoxicological data, through the computation of the Risk Quotient (RQ), using the Eq. (1).

1 $$RQ =\frac{MEC}{PNEC}$$

RQ is a ratio between exposure and effect where values < 1 indicate no risk, while RQ values ≥ 1 implies environmental risk^59,60^. Median Maximum Environmental Concentration (MEC) for NaCl occurring in aquifers of southeastern Australia were obtained from the available literature (8.259 NaCl g/L)^10^. Predicted No Effect Concentration (PNEC) values were estimated using a probabilistic approach from Species Sensitivity Distribution (SSD) modelling^61^. SSDs are used to estimate the level of a stressor that is protective for 95% of all species in the environment. This value is referred to as HC_5_^60,61^ and used here as a PNEC.

In the absence of chronic ecotoxicological data for groundwater organisms, SSD curves were computed using acute data from at least five different representative taxa^60,61^. We used the LC_50_ values generated in this study (for two primary consumers (copepods) and one predator (syncarid)), and LC_50_ for freshwater species from the U.S. EPA ECOTOX database (https://cfpub.epa.gov/ecotox/advanced_query.htm), as a surrogate for groundwater species^62^. Surface freshwater photosynthetic test organisms were not included because of their general absence from aphotic ecosystems^2^. Taxa included were oligochaetes, nematodes and crustaceans, which represent the fauna found in Australia groundwater ecosystems^63^. Only complete data records (values with symbols as > or < were discarded) corresponding to 48–96 h LC_50_ and EC_50_ values were extracted, giving 44 additional acute sensitivity data belonging to nine freshwater taxa (Supplementary Table S2). HC_5_ values were obtained by fitting a cumulative distribution to the ranked toxicity data using the CADDIS Volume 4: SSD Generator V1 spreadsheet provide by the US Environmental Protection Agency^64^.

## Supplementary information

Supplementary information.

## References

[1] Gaston L, Lapworth DJ, Stuart M, Amscheidt J (2019). Prioritization approaches for substances of emerging concern in groundwater: a critical review. Environ. Sci. Technol..

[2] Castaño-Sánchez A, Hose GC, Reboleira ASPS (2020). Ecotoxicological effects of anthropogenic stressors in subterranean organisms: a review. Chemosphere.

[3] Mammola S (2019). Scientists’ warning on the conservation of subterranean ecosystems. Bioscience.

[4] Foster SSD, Chilton PJ (2003). Groundwater: the processes and global significance of aquifer degradation. Philos. Trans. R. Soc. Lond. B. Biol. Sci..

[5] Masterson JP, Garabedian SP (2007). Effects of sea-level rise on ground water flow in a coastal aquifer system. Groundwater.

[6] Ferguson G, Gleeson T (2012). Vulnerability of coastal aquifers to groundwater use and climate change. Nat. Clim. Change.

[7] Robinson HK, Hasenmueller EA (2017). Transport of road salt contamination in karst aquifers and soils over multiple timescales. Sci. Total Environ..

[8] Davis J, Sim L, Chambers J (2010). Multiple stressors and regime shifts in shallow aquatic ecosystems in antipodean landscapes. Freshw. Biol..

[9] Davis J (2015). When trends intersect: the challenge of protecting freshwater ecosystems under multiple land use and hydrological intensification scenarios. Sci. Total Environ..

[10] Bennetts DA, Webb JA, Stone DJM, Hill DM (2006). Understanding the salinisation process for groundwater in an area of south-eastern Australia, using hydrochemical and isotopic evidence. J. Hydrol..

[11] Cartwright I, Weaver TR, Stone D, Reid M (2007). Constraining modern and historical recharge from bore hydrographs, 3H, 14C, and chloride concentrations: applications to dual-porosity aquifers in dryland salinity areas, Murray Basin, Australia. J. Hydrol..

[12] Bann, G. & Field, J. S. Dryland salinity, regolith and biodiversity: problems and opportunities for mitigation and remediation. *Proceedings of Regolith 2005—Ten Years of CRC LEME*, 8–12 (2005).

[13] National Land and Water Resources Audit (2001). A Summary of the National Land and Water Resources Audit’s ‘Australian Dryland Salinity Assessment 2000’ NLWRA.

[14] Velasco J (2018). Effects of salinity changes on aquatic organisms in a multiple stressor context. Philos. Trans. R. Soc. B. Biol. Sci..

[15] Di Lorenzo T, Galassi D (2017). Effect of temperature rising on the stygobitic crustacean species *Diacyclops belgicus*: does global warming affect groundwater populations?. Water.

[16] Addo-Bediako A, Chown SL, Gaston KJ (2000). Thermal tolerance, climatic variability and latitude. Proc. R. Soc. Lond. B Biol. Sci..

[17] Hughes L (2003). Climate change and Australia: trends, projections and impacts. Aust. Ecol..

[18] Badino G (2004). Cave temperatures and global climatic change. Int. J. Speleol..

[19] Griebler C (2010). Ecological assessment of groundwater ecosystems—vision or illusion?. Ecol. Eng..

[20] Griebler C, Avramov M (2015). Groundwater ecosystem services: a review. Freshw. Sci..

[21] Sket B (2018). Collecting and processing crustaceans of subterranean habitats. J. Crustacean. Biol..

[22] Hart RC, Bychek EA (2011). Body size in freshwater planktonic crustaceans: an overview of extrinsic determinants and modifying influences of biotic interactions. Hydrobiologia.

[23] Strong DR (1972). Life history variation among populations of an amphipod (*Hyalella azteca*). Ecology.

[24] Wong LC, Kwok KW, Leung KM, Wong CK (2009). Relative sensitivity distribution of freshwater planktonic crustaceans to trace metals. Hum. Ecol. Risk Assess..

[25] Hayasaka D, Korenaga T, Suzuki K, Sánchez-Bayo F, Goka K (2012). Differences in susceptibility of five cladoceran species to two systemic insecticides, imidacloprid and fipronil. Ecotoxicology.

[26] Sánchez-Bayo F (2006). Comparative acute toxicity of organic pollutants and reference values for crustaceans. I. Branchiopoda, Copepoda and Ostracoda. Environ. Pollut..

[27] Peck LS, Clark MS, Morley SA, Massey A, Rossetti H (2009). Animal temperature limits and ecological relevance: effects of size, activity and rates of change. Funct. Ecol..

[28] Nielsen DL, Brock MA, Rees GN, Baldwin DS (2003). Effects of increasing salinity on freshwater ecosystems in Australia. Aust. J. Bot..

[29] Menció A, Korbel KL, Hose GC (2014). River-aquifer interactions and their relationship to stygofauna assemblages: a case study of the Gwydir River alluvial aquifer (New South Wales, Australia). Sci. Total Environ..

[30] Shapouri M (2015). The variation of stygofauna along a gradient of salinization in a coastal aquifer. Hydrol. Res..

[31] Schulz C, Steward AL, Prior A (2013). Stygofauna presence within fresh and highly saline aquifers of the border rivers region in southern Queensland. Proc. Royal Soc. Qld..

[32] Reboleira ASPS, Abrantes NA, Oromí P, Gonçalves F (2013). Acute toxicity of copper sulfate and potassium dichromate on stygobiont *Proasellus*: general aspects of groundwater ecotoxicology and future perspectives. Water Air Soil Pollut..

[33] Peck LS, Morley SA, Richard J, Clark MS (2014). Acclimation and thermal tolerance in antarctic marine ectotherms. J. Exp. Biol..

[34] Issartel J, Hervant F, Voituron Y, Renault D, Vernon P (2005). Behavioural, ventilatory and respiratory responses of epigean and hypogean crustaceans to different temperatures. Comp. Biochem. Phys. A..

[35] Mermillod-Blondin F (2013). Thermal tolerance breadths among groundwater crustaceans living in a thermally constant environment. J. Exp. Biol..

[36] Terblanche JS, Deere JA, Clusella-Trullas S, Janion C, Chown SL (2007). Critical thermal limits depend on methodological context. Proc. R. Soc. B Biol. Sci..

[37] Chown SL, Jumbam KR, Sørensen JG, Terblanche JS (2009). Phenotypic variance, plasticity and heritability estimates of critical thermal limits depend on methodological context. Funct. Ecol..

[38] Verberk WCEP (2016). Does oxygen limit thermal tolerance in arthropods? A critical review of current evidence. Comp. Biochem. Phys. A..

[39] Zhu K, Grathwohl P (2015). Groundwater temperature evolution in the subsurface urban heat island of Cologne, Germany. Hydrol. Process..

[40] Griebler C (2016). Potential impacts of geothermal energy use and storage of heat on groundwater quality, biodiversity, and ecosystem processes. Environ. Earth Sci..

[41] Reid AJ (2019). Emerging threats and persistent conservation challenges for freshwater biodiversity. Biol. Rev..

[42] Juan C, Guzik MT, Jaume D, Cooper SJ (2010). Evolution in caves: Darwin’s ‘wrecks of ancient life’ in the molecular era. Mol. Ecol..

[43] Song MY, Brown JJ (1998). Osmotic effects as a factor modifying insecticide toxicity on *Aedes* and *Artemia*. Ecotox. Environ. Safe..

[44] Wang J, Grisle S, Schlenk D (2001). Effects of salinity on Aldicarb toxicity in juvenile rainbow trout (*Oncorhynchus mykiss*) and striped bass (*Morone saxatilis* x *chrysops*). Toxicol. Sci..

[45] Cairns J, Heath AG, Parker BC (1975). The effects of temperature upon the toxicity of chemicals to aquatic organisms. Hydrobiologia.

[46] Schiedek D, Sundelin B, Readman JW, Macdonald RW (2007). Interactions between climate change and contaminants. Mar. Pollut. Bull..

[47] Hose GC, Symington K, Lott M, Lategan M (2016). The toxicity of arsenic (III), chromium (VI) and zinc to groundwater copepods. Environ. Sci. Pollut. Res..

[48] Asmyhr MG, Hose GC, Graham P, Stow A (2014). Fine-scaled genetics of subterranean syncarids. Freshw. Biol..

[49] Galassi DM, Huys R, Reid JW (2009). Diversity, ecology and evolution of groundwater copepods. Freshw. Biol..

[50] Schminke HK, Cho JL (2013). Biology and ecology of Parabathynellidae (Crustacea, Bathynellacea)—a review. Crustaceana.

[51] ASTM (American Society for Testing and Materials). Standard guide for *Daphnia magna* life-cycle toxicity tests. Annual Book of ASTM Standards, Report E1193–97. (Philadelphia, USA, 1997).

[52] ISO (Internacional Organization for Standardization). Water quality: determination of the inhibition of the mobility of *Daphnia magna* Straus (Cladocera, Crustacea)—acute toxicity test. ISO 6341 (Geneva 1996).

[53] OECD (Organization for the Economic Cooperation and Development). Guideline for testing of chemicals *Daphnia* sp., Acute Immobilisation Test. OECD test guideline 202. (Paris, 2004).

[54] Di Lorenzo T (2019). Recommendations for ecotoxicity testing with stygobiotic species in the framework of groundwater environmental risk assessment. Sci. Total Environ..

[55] Rizzo V, Sánchez-Fernández D, Fresneda J, Cieslak A, Ribera I (2015). Lack of evolutionary adjustment to ambient temperature in highly specialized cave beetles. Evol. Biol..

[56] Ritz, C. & Streibig, J. C. Bioassay for allelochemicals: examples with RJ Stat. Software (2016).

[57] Ripley, B. D. & Venables, W. N. Feed-forward neural networks and multinomial log-linear models. R package version 7.3–12. (2018).

[58] Team R (2013). R Development core team. R. A. Lang. Environ. Stat. Comput..

[59] EMA (European Medicines Agency). Guidelines on the Environmental Risk Assessment of Medicinal Products for Human Use. Doc. Ref. 627 Risks of Veterinary Medicinal Products in Groundwater (2006).

[60] EC (European Commission). Technical Guidance Document in Support of Commission Directive 93/67/EEC on Risk Assessment for New Notified Substances and Commission Regulation (EC) N. 1488/94 on Risk Assessment for Existing Substances. Office for official publications of the European communities. (Luxembourg, 2003).

[61] EC (European Commission). Common Implementation Strategy for the Water Directive (2000/60/EC). Technical Guidance Document for Deriving Environmental Quality Standards. Technical Report 055 (2011).

[62] Hose GC (2005). Assessing the need for groundwater quality guidelines for pesticides using the species sensitivity distribution approach. Hum. Ecol. Risk. Assess..

[63] Hose GC, Asmyhr MG, Cooper SJB, Humphreys WF, Stow A, Maclean N, Holwell GI (2015). Down Under Down Under: Austral Groundwater Life. Austral Ark.

[64] USEPA. CADDIS Volume 4: SSD Generator V1. Available at https://www.epa.gov/caddis-vol4/caddis-volume-4-data-analysis-download-software#tab-3. Accessed 4 Feb 2020.

