# Supplementary material for: Salinity and temperature increase impact groundwater crustaceans
Source: Sci Rep. 2020 Jul 23;10:12328. doi: 10.1038/s41598-020-69050-7 (PMC7378218; doi:10.1038/s41598-020-69050-7)
Supplement: Supplementary file 1 — Supplementary information. [file 41598_2020_69050_MOESM1_ESM.docx]

**Supplementary Information:**

**Salinity and temperature increase impact groundwater crustaceans**

**Andrea Castaño-Sánchez^1^; Grant C Hose^2^; Ana Sofia P.S. Reboleira^1^ ^*^**

^1^ Natural History Museum of Denmark, University of Copenhagen, Universitetsparken 15, 2100 Copenhagen, Denmark

^2^ Department of Biological Sciences, Macquarie University, NSW 2109 Sydney, Australia

^*^sreboleira@snm.ku.dk

**Supplementary Table S.1:** Parameters and hazardous concentrations for the species sensitivity distribution curve fitted with 48-96 h LC_50_ NaCl values for freshwater invertebrates.

| PARAMETERS | |
| --- | --- |
| Slope | 2.64648051 |
| Intercept | 3.5539067 |
| R2 | 0.92345507 |
| GrandMean | 0.54642129 |
| SumSQ | 5.00557549 |
| CSSQ | 1.42266072 |
| MSE | 0.08259227 |
| Tcrit | 1.81246112 |
| N | 12 |
| df | 10 |

| **Protective concentration to different proportion of the species (hazardous concentrations)** | | | | | | | | |
| --- | --- | --- | --- | --- | --- | --- | --- | --- |
| **Proportion** | **Probit** | **Log Central Tendency** | **SSQ** | **Log Upper PI** | **Log Lower PI** | **Central Tendency** | **Upper PI** | **Lower PI** |
| 0.05 | 3.355 | -0.075 | 0.016 | 0.154 | -0.304 | 0.841 | 1.426 | 0.496 |
| 0.1 | 3.718 | 0.062 | 0.015 | 0.282 | -0.158 | 1.154 | 1.915 | 0.695 |
| 0.2 | 4.158 | 0.228 | 0.014 | 0.440 | 0.017 | 1.692 | 2.753 | 1.040 |
| 0.4 | 4.747 | 0.451 | 0.013 | 0.656 | 0.245 | 2.823 | 4.531 | 1.759 |
| 0.5 | 5.000 | 0.546 | 0.013 | 0.751 | 0.342 | 3.519 | 5.640 | 2.196 |
| 0.7 | 5.524 | 0.745 | 0.013 | 0.952 | 0.537 | 5.554 | 8.954 | 3.444 |
| 0.8 | 5.842 | 0.864 | 0.014 | 1.076 | 0.653 | 7.319 | 11.910 | 4.497 |
| 0.9 | 6.282 | 1.031 | 0.015 | 1.251 | 0.811 | 10.732 | 17.806 | 6.468 |
| 0.95 | 6.645 | 1.168 | 0.016 | 1.397 | 0.939 | 14.721 | 24.948 | 8.687 |

**Supplementary Table S.2:** Lethal NaCl concentration values affecting 50% of test populations (LC_50_) for freshwater crustaceans tested under static laboratory conditions in freshwater with 48-96 h exposure (obtained from the U.S. EPA ECOTOX database).

| **CAS Number** | **Chemical Name** | **Species Scientific Name** | **Species Group** | **Organism Lifestage** | **Exposure Type** | **Media Type** | **Test Location** | **Concentration (g/L) (Standardized)** | **Effect Measurement** | **Endpoint** | **Observed Duration (Days)** | **Reference** |
| --- | --- | --- | --- | --- | --- | --- | --- | --- | --- | --- | --- | --- |
| 7647145 | Sodium chloride (NaCl) | *Asellus communis* | Crustaceans | Not reported | Static | Fresh water | Lab | 5.1 | Mortality | LC50 | 4 | ^1^ |
| 7647145 | Sodium chloride (NaCl) | *Asellus communis* | Crustaceans | Not reported | Static | Fresh water | Lab | 8.25 | Mortality | LC50 | 4 | ^1^ |
| 7647145 | Sodium chloride (NaCl) | *Caenorhabditis elegans* | Worms; Standard Test Species | Adult | Static | Fresh water | Lab | 14.899 | Mortality | LC50 | 2 | ^2^ |
| 7647145 | Sodium chloride (NaCl) | *Caenorhabditis elegans* | Worms; Standard Test Species | Adult | Static | Fresh water | Lab | 24.829 | Mortality | LC50 | 2 | ^2^ |
| 7647145 | Sodium chloride (NaCl) | *Caenorhabditis elegans* | Worms; Standard Test Species | Adult | Static | Fresh water | Lab | 22.457 | Mortality | LC50 | 2 | ^2^ |
| 7647145 | Sodium chloride (NaCl) | *Ceriodaphnia dubia* | Crustaceans; Standard Test Species | Neonate | Static | Fresh water | Lab | 1.356 | Mortality | LC50 | 2 | ^3^ |
| 7647145 | Sodium chloride (NaCl) | *Ceriodaphnia dubia* | Crustaceans; Standard Test Species | Neonate | Static | Fresh water | Lab | 1.249 | Mortality | LC50 | 2 | ^3^ |
| 7647145 | Sodium chloride (NaCl) | *Ceriodaphnia dubia* | Crustaceans; Standard Test Species | Neonate | Static | Fresh water | Lab | 1.154 | Mortality | LC50 | 2 | ^3^ |
| 7647145 | Sodium chloride (NaCl) | *Ceriodaphnia dubia* | Crustaceans; Standard Test Species | Neonate | Static | Fresh water | Lab | 1.836 | Mortality | LC50 | 2 | ^3^ |
| 7647145 | Sodium chloride (NaCl) | *Ceriodaphnia dubia* | Crustaceans; Standard Test Species | Not reported | Static | Fresh water | Lab | 1.96 | Mortality | LC50 | 2 | ^4^ |
| 7647145 | Sodium chloride (NaCl) | *Ceriodaphnia dubia* | Crustaceans; Standard Test Species | Not reported | Static | Fresh water | Lab | 1.068 | Mortality | LC50 | 2 | ^5^ |
| 7647145 | Sodium chloride (NaCl) | *Ceriodaphnia dubia* | Crustaceans; Standard Test Species | Neonate | Static | Fresh water | Lab | 1.489 | Mortality | LC50 | 2 | ^3^ |
| 7647145 | Sodium chloride (NaCl) | *Ceriodaphnia dubia* | Crustaceans; Standard Test Species | Neonate | Static | Fresh water | Lab | 1.779 | Mortality | LC50 | 2 | ^3^ |
| 7647145 | Sodium chloride (NaCl) | *Ceriodaphnia dubia* | Crustaceans; Standard Test Species | Neonate | Static | Fresh water | Lab | 0.861 | Mortality | LC50 | 2 | ^3^ |
| 7647145 | Sodium chloride (NaCl) | *Ceriodaphnia dubia* | Crustaceans; Standard Test Species | Neonate | Static | Fresh water | Lab | 1.402 | Mortality | LC50 | 2 | ^3^ |
| 7647145 | Sodium chloride (NaCl) | *Ceriodaphnia dubia* | Crustaceans; Standard Test Species | Neonate | Static | Fresh water | Lab | 1.589 | Mortality | LC50 | 2 | ^3^ |
| 7647145 | Sodium chloride (NaCl) | *Ceriodaphnia dubia* | Crustaceans; Standard Test Species | Not reported | Static | Fresh water | Lab | 1.042 | Mortality | LC50 | 2 | ^6^ |
| 7647145 | Sodium chloride (NaCl) | *Ceriodaphnia dubia* | Crustaceans; Standard Test Species | Neonate | Static | Fresh water | Lab | 1.59 | Mortality | LC50 | 2 | ^7^ |
| 7647145 | Sodium chloride (NaCl) | *Ceriodaphnia dubia* | Crustaceans; Standard Test Species | Neonate | Static | Fresh water | Lab | 1.192 | Mortality | LC50 | 2 | ^3^ |
| 7647145 | Sodium chloride (NaCl) | *Ceriodaphnia dubia* | Crustaceans; Standard Test Species | Neonate | Static | Fresh water | Lab | 1.357 | Mortality | LC50 | 2 | ^3^ |
| 7647145 | Sodium chloride (NaCl) | *Ceriodaphnia dubia* | Crustaceans; Standard Test Species | Neonate | Static | Fresh water | Lab | 1.317 | Mortality | LC50 | 2 | ^3^ |
| 7647145 | Sodium chloride (NaCl) | *Ceriodaphnia dubia* | Crustaceans; Standard Test Species | Neonate | Static | Fresh water | Lab | 0.977 | Mortality | LC50 | 2 | ^3^ |
| 7647145 | Sodium chloride (NaCl) | *Ceriodaphnia dubia* | Crustaceans; Standard Test Species | Not reported | Static | Fresh water | Lab | 4.7 | Mortality | LC50 | 2 | ^8^ |
| 7647145 | Sodium chloride (NaCl) | *Daphnia ambigua* | Crustaceans | Neonate | Static | Fresh water | Lab | 2 | Mortality | LC50 | 2 | ^7^ |
| 7647145 | Sodium chloride (NaCl) | *Daphnia magna* | Crustaceans; Standard Test Species | Not reported | Static | Fresh water | Lab | 4.77 | Mortality | LC50 | 2 | ^4^ |
| 7647145 | Sodium chloride (NaCl) | *Daphnia magna* | Crustaceans; Standard Test Species | Neonate | Static | Fresh water | Lab | 5.48 | Mortality | LC50 | 2 | ^9^ |
| 7647145 | Sodium chloride (NaCl) | *Daphnia magna* | Crustaceans; Standard Test Species | Not reported | Static | Fresh water | Lab | 3.31 | Mortality | LC50 | 2 | ^10^ |
| 7647145 | Sodium chloride (NaCl) | *Daphnia magna* | Crustaceans; Standard Test Species | Neonate | Static | Fresh water | Lab | 6.027 | Mortality | LC50 | 2 | ^11^ |
| 7647145 | Sodium chloride (NaCl) | *Daphnia magna* | Crustaceans; Standard Test Species | Neonate | Static | Fresh water | Lab | 5.6 | Mortality | LC50 | 2 | ^11^ |
| 7647145 | Sodium chloride (NaCl) | *Daphnia magna* | Crustaceans; Standard Test Species | Neonate | Static | Fresh water | Lab | 5.6 | Mortality | LC50 | 2 | ^11^ |
| 7647145 | Sodium chloride (NaCl) | *Daphnia magna* | Crustaceans; Standard Test Species | Multiple | Static | Fresh water | Lab | 3.318 | Mortality | LC50* | 2 | ^12^ |
| 7647145 | Sodium chloride (NaCl) | *Daphnia magna* | Crustaceans; Standard Test Species | Neonate | Static | Fresh water | Lab | 6.027 | Mortality | LC50 | 2 | ^11^ |
| 7647145 | Sodium chloride (NaCl) | *Daphnia magna* | Crustaceans; Standard Test Species | Not reported | Static | Fresh water | Lab | 3.137 | Mortality | LC50 | 2 | ^13^ |
| 7647145 | Sodium chloride (NaCl) | *Daphnia magna* | Crustaceans; Standard Test Species | Neonate | Static | Fresh water | Lab | 5.02 | Mortality | LC50 | 2 | ^11^ |
| 7647145 | Sodium chloride (NaCl) | *Daphnia magna* | Crustaceans; Standard Test Species | Not reported | Static | Fresh water | Lab | 3.136 | Mortality | LC50 | 2 | ^13^ |
| 7647145 | Sodium chloride (NaCl) | *Daphnia magna* | Crustaceans; Standard Test Species | Not reported | Static | Fresh water | Lab | 1.56 | Mortality | LC50 | 2 | ^8^ |
| 7647145 | Sodium chloride (NaCl) | *Daphnia magna* | Crustaceans; Standard Test Species | Not reported | Static | Fresh water | Lab | 3.63 | Mortality | LC50 | 2 | ^5^ |
| 7647145 | Sodium chloride (NaCl) | *Daphnia magna* | Crustaceans; Standard Test Species | Not reported | Static | Fresh water | Lab | 3.22 | Mortality | LC50 | 2 | ^13^ |
| 7647145 | Sodium chloride (NaCl) | *Daphnia pulex* | Crustaceans; Standard Test Species | Neonate | Static | Fresh water | Lab | 3.32 | Mortality | LC50 | 2 | ^14^ |
| 7647145 | Sodium chloride (NaCl) | *Daphnia pulex* | Crustaceans; Standard Test Species | Not reported | Static | Fresh water | Lab | 3.05 | Mortality | LC50 | 2 | ^15^ |
| 7647145 | Sodium chloride (NaCl) | *Daphnia pulex* | Crustaceans; Standard Test Species | Neonate | Static | Fresh water | Lab | 4.05 | Mortality | LC50 | 2 | ^14^ |
| 7647145 | Sodium chloride (NaCl) | *Daphnia pulex* | Crustaceans; Standard Test Species | Not reported | Static | Fresh water | Lab | 1.47 | Mortality | LC50 | 2 | ^15^ |
| 7647145 | Sodium chloride (NaCl) | *Gammarus pseudolimnaeus* | Crustaceans; Standard Test Species | Not reported | Static | Fresh water | Lab | 7.7 | Mortality | LC50 | 4 | ^16^ |
| 7647145 | Sodium chloride (NaCl) | *Hyalella azteca* | Crustaceans; Standard Test Species | Not reported | Static | Fresh water | Lab | 1.382 | Mortality | LC50 | 4 | ^5^ |

**Supplementary table S.3:** Treatment concentrations used in the acute NaCl test and average dissolved oxygen (DO), concentration measured at the beginning (T0) and end (T96h) of the experiment.

| **Taxa** | **NaCl concentration (g/L)** | **T0 DO (mg/L)** | **T96h DO (mg/L)** |
| --- | --- | --- | --- |
| Cyclopoida | control | 5.97 | 5.65 |
| Cyclopoida | 2 | 5.7 | 5.9 |
| Cyclopoida | 2.6 | 5.58 | 5.55 |
| Cyclopoida | 3.5 | 5.95 | 5.5 |
| Cyclopoida | 4.7 | 5.53 | 5.4 |
| Cyclopoida | 6.25 | 5.67 | 5.65 |
| Cyclopoida | 8.32 | 5.29 | 5.45 |
| Harpaticoida | control | 6.25 | 7 |
| Harpaticoida | 0.37 | 6.8 | 6.95 |
| Harpaticoida | 0.7 | 6.85 | 6.75 |
| Harpaticoida | 1.33 | 6.9 | 6.9 |
| Harpaticoida | 2.52 | 6.85 | 6.55 |
| Harpaticoida | 4.8 | 7 | 6.95 |
| Harpaticoida | 9.12 | 7 | 7.3 |
| Syncarida | control | 5.55 | 5.1 |
| Syncarida | 5.5 | 5.86 | 5.6 |
| Syncarida | 6.87 | 5.8 | 5.35 |
| Syncarida | 8.5 | 6.33 | 5.9 |
| Syncarida | 10.78 | 5.55 | 5.5 |
| Syncarida | 13.42 | 5.72 | 5.8 |
| Syncarida | 16.78 | 5.365 | 6.3 |

**Supplementary references:**

1. Wurtz, C. B. & Bridges, C. H. Preliminary Results From Macro-Invertebrate Bioassays. *Proc. Pa. Acad. Sci.* 35, 51-56 (1961).
2. Cressman C. P. & Williams, P. L. Reference Toxicants for Toxicity Testing Using *Caenorhabditis elegans* in Aquatic Media. In Environmental Toxicology and Risk Assessment: Modeling and Risk Assessment Sixth Volume (ed. Dwyer, F. Doane, T. & Hinman, M.) 518-532 (West Conshohocken, PA: ASTM International, 1997).
3. Soucek, D. J. *et al.* Influence of Water Hardness and Sulfate on the Acute Toxicity of Chloride to Sensitive Freshwater Invertebrates*. Environ. Toxicol. Chem.* 30(4), 930-938 (2011).
4. Mount, D. R., Gulley, D. D., Hockett, J. R., Garrison, T. D. & Evans, J. M. Statistical Models to Predict the Toxicity of Major Ions to *Ceriodaphnia dubia, Daphnia magna* and *Pimephales promelas* (Fathead Minnows). *Environ. Toxicol. Chem.* 16(10), 2009-2019 (1997).
5. Elphick, J. R. F., Bergh, K. D. & Bailey, H. C. Chronic Toxicity of Chloride to Freshwater Species: Effects of Hardness and Implications for Water Quality Guidelines. *Environ. Toxicol. Chem.* 30(1), 239-246 (2011).
6. Mount, D. R. & Gulley, D. D. Development of a Salinity/Toxicity Relationship to Predict Acute Toxicity of Saline Waters to Freshwater Organisms. Interim final report, June 1990- March 1992. ENSR Consulting and Engineering, Fort Collings, CO (United States, 1992).
7. Harmon, S. M., Specht, W. L. & Chandler, G. T. A Comparison of the Daphnids *Ceriodaphnia dubia* and *Daphnia ambigua* for Their Utilization in Routine Toxicity Testing in the Southeastern United States. *Arch. Environ. Contam. Toxicol.* 45(1), 79-85 (2003).
8. Kszos, L. A., Talmage, S. S., Morris, G. W., Konetsky, B. K. & Rottero, T. Derivation of Aquatic Screening Benchmarks for 1,2-Dibromoethane. *Arch. Environ. Contam. Toxicol.* 45(1), 66-71 (2003).
9. Martinez-Jeronimo, F. & Martinez-Jeronimo, L. Chronic Effect of NaCl Salinity on a Freshwater Strain of *Daphnia magna Straus* (Crustacea: Cladocera): A Demographic Study. *Ecotoxicol. Environ. Saf.* 67(3), 411-416 (2007).
10. Dowden, B. F. & Bennett, H. J. Toxicity of Selected Chemicals to Certain Animals. *J. Water Pollut. Control Fed.* 37(9), 1308-1316. (1965).
11. Harris, G. The Comparative Toxicity of Crude and Refined Oils to *Daphnia magna*. Environment Canada, Environmental Technology Centre, Emergencies Science Division. (Ottawa, Canada, 1994).
12. Dowden, B. F. Cumulative Toxicities of Some Inorganic Salts to *Daphnia magna* as Determined by Median Tolerance Limits. *Proc. La. Acad. Sci.* 23, 77-85 (1961).
13. Davies, T. D. & Hall, K. J. Importance of Calcium in Modifying the Acute Toxicity of Sodium Sulphate to *Hyalella azteca* and *Daphnia magna*. *Environ. Toxicol. Chem.* 26(6), 1243-1247 (2007).
14. Bezirci, G. *et al*. Impacts of Salinity and Fish-Exuded Kairomone on the Survival and Macromolecular Profile of *Daphnia pulex*. *Ecotoxicology* 21, 601-614 (2012).
15. Birge, W. J. *et al.* Recommendations on Numerical Values for Regulating Iron and Chloride Concentrations for the Purpose of Protecting Warmwater Species of Aquatic Life in the Commonwealth of Kentucky (University of Kentucky, Lexington, 1985).
16. Blasius, B. J. & Merritt, R. W. Field and Laboratory Investigations on the Effects of Road Salt (NaCl) on Stream Macroinvertebrate Communities. *Environ. Pollut.* 120(2), 219-231 (2002).
